# A public health approach to optimizing surgical outcomes for hepatic hydatid cyst: identifying predictive factors for postoperative morbidity

**DOI:** 10.3389/fpubh.2026.1926824

**Published:** 2026-09-03

**Authors:** Jihen Krichen, Asma Sghaier, Maissa Timoumi, Saif Farhaoui, Sihem Sindi, Bilel Faidi, Khalil Ben Salah, Hajer Hannachi, Latifa Merzougui

**Affiliations:** 1 Digestive and Visceral Surgery Department, Aghlabides Surgical Unit, Ibn Aljazzar Hospital, Kairouan, Tunisia; 2 Digestive and Visceral Surgery Department, Farhat Hached University Hospital, Sousse, Tunisia; 3 Department of Preventive Medicine, Ibn Aljazzar University Hospital, Kairouan, Tunisia; 4 Faculty of Medicine of Sousse, University of Sousse, Sousse, Tunisia

**Keywords:** biliary fistula, echinococcosis, hepatic hydatid cyst, postoperative morbidity, public health, risk management, surgical outcomes

## Abstract

**Background:**

Hepatic hydatid cyst (HHC) remains a significant public health challenge in endemic regions, causing substantial morbidity and placing a persistent burden on healthcare systems. While surgery is the primary therapeutic intervention, the high rate of specific postoperative complications, particularly biliary fistulas, necessitates a more strategic approach to clinical risk management and patient stratification to optimize resource utilization. This study aims to identify predictive factors for postoperative complications to guide clinical decision-making and enhance the efficiency of surgical care in endemic settings.

**Methods:**

We conducted a retrospective cohort study of 107 patients (168 cysts) who underwent surgery for HHC at the Ibn Al Jazzar University Hospital, Kairouan, over an 8-year period (2017–2025). We analyzed a comprehensive set of clinical, biological, and radiological data using univariate and multivariate binary logistic regression models to isolate independent predictors of specific postoperative morbidity.

**Results:**

Conservative surgical management was employed in 89.7% of patients. The overall postoperative complication rate was 15.9%, with biliary fistula identified as the most frequent specific complication (7.5%). Multivariate analysis confirmed that suspected complications on preoperative CT scans (*p* = 0.03) and the presence of intraoperative complications (*p* < 0.01) were independent, significant predictive factors for specific postoperative morbidity.

**Conclusion:**

Preoperative CT imaging is a robust, non-invasive triage tool for identifying high-risk patients, which is critical for health system sustainability. Integrating these predictive markers into clinical protocols will allow for optimized surgical planning, reduced hospital resource consumption, and the mitigation of the public health burden associated with HHC in endemic regions.

## 1 Introduction

Cystic echinococcosis, caused by the larval stage of the cestode *Echinococcus granulosus*, is a major zoonotic disease with a global, yet uneven, distribution (1). In endemic areas, including the Mediterranean basin, the Middle East, Latin America, and China, the disease poses a significant public health challenge (1). In Tunisia, hepatic hydatid cyst (HHC) remains a persistent endemic condition, imposing a substantial burden on healthcare systems due to its high prevalence and the constant demand for specialized surgical resources (2).

Although surgery is considered the gold standard for eradicating the parasite and managing the residual cavity, it is frequently associated with high morbidity rates (3). This postoperative morbidity, which significantly contributes to prolonged hospital stays and increased resource utilization, is primarily driven by the occurrence of biliary fistulas (4).

The clinical debate regarding the optimal surgical approach—conservative versus radical—remains ongoing (3). While conservative surgery is technically more accessible, it is frequently linked to elevated postoperative complication rates compared to radical procedures (4). To date, predictive factors for these complications are not well-established, suggesting that clinical evaluations should avoid overly rigid assertions when assessing individual patient risks (1, 3).

In this context, optimizing clinical management by identifying predictive risk factors at the preoperative stage is essential to reduce preventable morbidity in endemic regions (1, 4). The main objective of this study was to identify the predictive factors for postoperative complications following HHC surgery, avoiding categorical assertions while providing evidence-based indicators. This research aims to provide clear clinical and radiological indicators to improve surgical planning and the allocation of hospital resources in areas where echinococcosis remains a significant public health burden (1, 2).

## 2 Materials and methods

### 2.1 Study design and setting

We conducted a retrospective cohort study at the General Surgery Department of the Ibn Al Jazzar University Hospital, Kairouan, Tunisia, spanning an 8-year period from January 1, 2017, to September 31, 2025. This study protocol was designed to evaluate clinical and radiological predictive factors for postoperative morbidity in patients undergoing surgery for hepatic hydatid cysts (HHC). The cyst was used as the unit of analysis; for patients with multiple cysts, each was considered an independent entity to appropriately capture localized cyst-specific morphological risks for complications.

### 2.2 Inclusion and exclusion criteria

To ensure the robustness of our analysis and the homogeneity of the study population, the following criteria were applied:

- Inclusion Criteria:

- All patients who underwent elective or emergency surgical intervention for HHC at our institution during the study period.
- Patients with HHC regardless of the number, anatomical location, size, or degree of cyst complexity.

- Non-Inclusion and Exclusion Criteria:

- Patients with incomplete medical records, rendering the extraction of key clinical, radiological, or surgical variables impossible.
- Patients managed exclusively via the Puncture, Aspiration, Injection, and Re-aspiration (PAIR) technique, as this study focuses specifically on surgical outcomes and the management of the residual cavity.
- Patients who received medical treatment for HHC without subsequent surgical intervention.

### 2.3 Data collection and variables

Medical records were systematically reviewed to extract comprehensive patient data, including demographic characteristics (age, sex, and rural/urban residence), history of hydatid exposure, and clinical presentation. Biological data, including preoperative liver function tests and hydatid serology, were also recorded. Radiological findings were retrieved from ultrasound, CT scans, and MRI, documenting cyst morphology according to the WHO (World Health Organization) ultrasound classification (5), localization, and the presence of preoperative complications such as biliary-cystic fistulas (defined as > 5 mm for large fistulas). Data parameters and metrics underwent rigorous review to ensure precision and accuracy.

### 2.4 Statistical analysis

The cyst was used as the unit of analysis; for patients with multiple cysts, each was considered an independent entity.

- Qualitative variables were expressed as frequencies and percentages.
- Quantitative variables were tested for normal distribution using Shapiro–Wilk and Kolmogorov–Smirnov tests. Gaussian variables were presented as mean and standard deviation, while non-Gaussian variables were presented as median and interquartile range (IQR).
- For univariate analysis, we utilized the Chi-square test for categorical variables and the t-test or Mann–Whitney U test for continuous variables.
- Variables with a *p* < 0.20 in univariate analysis were entered into a stepwise descending binary logistic regression model. Significance was established at *p* < 0.05.
- Data entry and analysis were performed using SPSS version 26.0.

## 3 Results

### 3.1 Descriptive analysis

#### 3.1.1 Patient population and epidemiological characteristics

**Figure 1:**
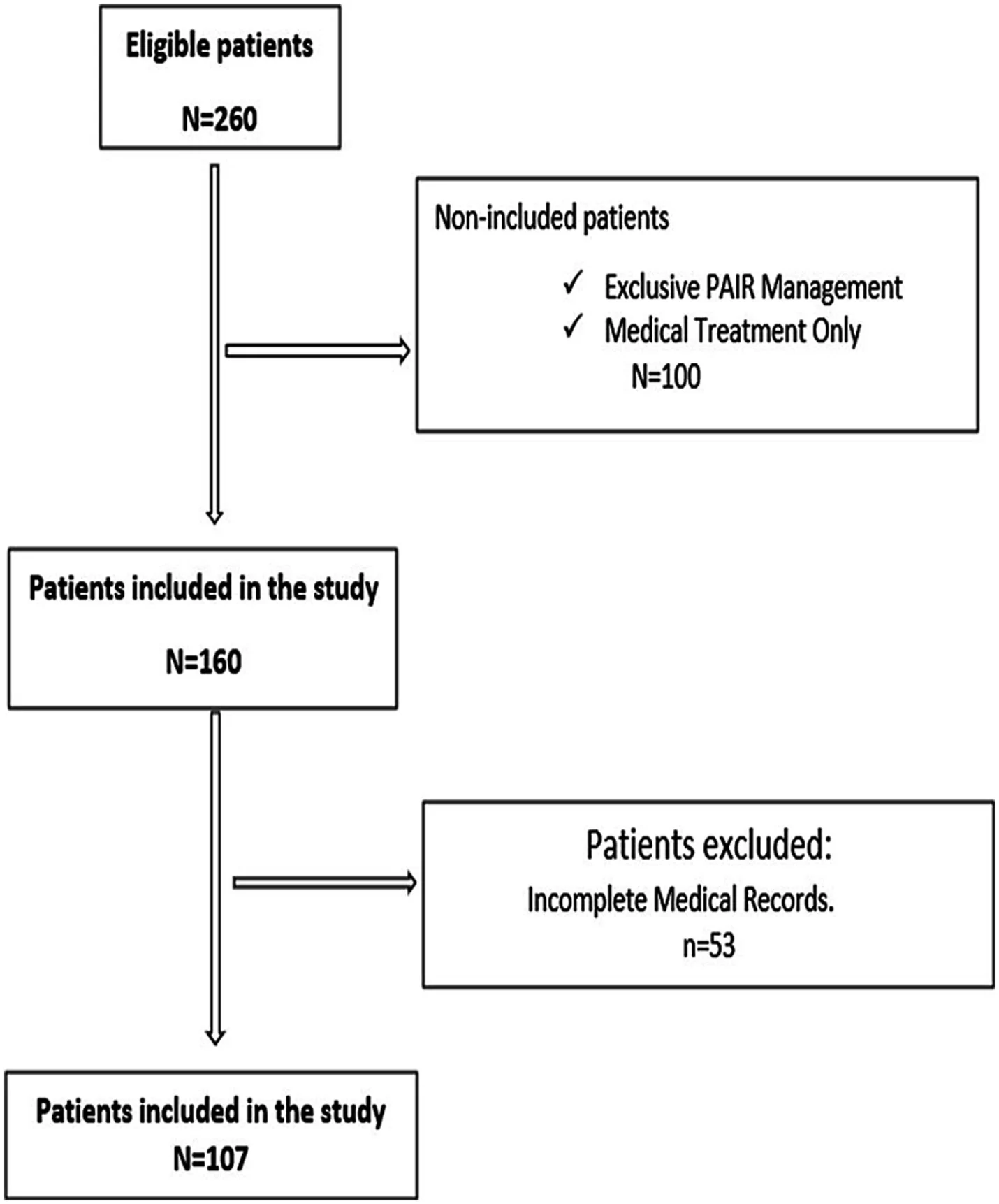
Flow diagram.

This study included a total of 107 patients who underwent surgical intervention for hepatic hydatid cysts (HHC) at our institution. The selection process included all eligible patients after strict application of inclusion and exclusion criteria, as detailed in Figure 1. The selection process included all patients who underwent elective or emergency surgical procedures for HHC, regardless of cyst number, anatomical location, size, or degree of complexity. The exclusion criteria strictly excluded patients with incomplete medical records that rendered the extraction of clinical, radiological, or surgical variables impossible. Furthermore, patients managed exclusively via the Puncture, Aspiration, Injection, and Re-aspiration (PAIR) technique, as well as those who received medical treatment without subsequent surgical intervention, were excluded from the analysis. (Figure 1).

A total of 168 cysts were identified. Key demographic and clinical characteristics are summarized in Table 1.

- Demographics: The median age was 39.5 years (IQR = 27–58).
- Gender Distribution: There was a female predominance with 65 patients (61%), resulting in a sex ratio of 0.64.
- Epidemiological Context: A majority of patients (84.6%) resided in rural areas. A history of contact with dogs and livestock was reported by 70 patients (65.4%).

**Table 1:** Characteristics of hydatid cysts on abdominal CT scan.

Characteristic	Subgroup	Number (*N* = 150)
Size	<10 cm ≥ 10 cm	126 24
Segments	Segment IV Segment VI Segment VII Segment VIII	22 28 29 25
Complications	Intrabiliary rupture Vascular compression Suppuration	15 9 2

#### 3.1.2 Clinical and biological findings

- Clinical Presentation: Abdominal pain in the right hypochondrium was the most frequent presenting symptom (86%), while 14% of cases were discovered incidentally.
- Laboratory Data: Hydatid serology was positive in 60.7% of the 78 patients tested. Among those who underwent liver function tests, 14.9% exhibited signs of cholestasis.

#### 3.1.3 Imaging findings

- Localization: Ultrasound identified 122 cysts, predominantly located in segments VI (22%) and VII (18%).
- Cyst Dimensions: The median cyst diameter was 68 mm (range: 10–180 mm).
- Classification: According to the OMS classification (detailed in Table 2), types III and IV were the most prevalent, each accounting for 29.2% of the cysts.

**Table 2:** Specific surgical postoperative complications in patients operated on for hepatic hydatid cyst.

Complications	N	(%)
Non-specific morbidity	7	6.5
*Medical*	6	5.6
*Surgical*	1	0.9
Specific surgical morbidity	10	9.3
*Biliary fistula*	8	7.5
*Suppuration of the residual cavity*	1	0.9
*Purulent retention*	1	0.9

#### 3.1.4 Surgical management and postoperative outcomes

- Surgical Approach: All patients underwent laparotomy. The surgical procedures performed are detailed in Table 3. A conservative surgical approach was performed in 89.7% (*n* = 96) of patients, with the resection of the prominent dome performed in 96.7% of these cases.
- Morbidity: Postoperative recovery was uneventful in 84.1% (*n* = 90) of patients. Postoperative complications occurred in 17 patients (15.9%). The evaluated tables confirm that the total patient cohort consists precisely of 107 individuals. The distribution of these complications is presented in Table 4.

- Specific Complications: Observed in 9.3% (*n* = 10) of patients, primarily driven by biliary fistulas (7.5%). (7.5%, corresponding to 8 patients out of the 107 total).
- Non-Specific Complications: Occurred in 6.5% (*n* = 7) of patients.
- Follow-up: The recurrence rate after 3 years of follow-up was 11.2%.

**Table 3:** Postoperative complications following conservative surgical treatment.

Morbidity	*n* (%)
Non-specific morbidity	**7 (6.5)**
*Medical*	6 (5.6)
*Surgical*	1 (0.9)
Specific surgical morbidity	**10 (9.3)**
*Biliary fistula*	8 (7.5)
*Suppuration of the residual cavity*	1 (0.9)
*Purulent retention*	1 (0.9)

Bold values indicate data from the present study.

**Table 4:** Association between cyst characteristics and specific postoperative complications.

Cyst characteristics	Category/Subgroup	*n* (%)	*p*-value	OR	95% CI
Ultrasound-detected complication	Yes (*n* = 11) No (*n* = 68)	3 (3.8) 3 (3.8)	**0.033**	8.12	1.397–47.262
CT-detected complication	Yes (*n* = 27) No (*n* = 73)	6 (6.0) 3 (3.0)	**0.011**	6.66	1.534–28.972
WHO classification	CE2/CE3 (*n* = 28) CE4 (*n* = 20)	2 (2.5)	1	-	-
		3 (3.8)	0.16	3.29	0.608–17.849
Cyst size	≥ 10 cm (*n* = 31) < 10 cm (*n* = 69)	4 (4.0) 3 (3.0)	0.13	3.12	0.777–12.562
Calcifications	Yes (*n* = 39) No (*n* = 61)	2 (2.0) 7 (7.0)	0.47	-	-
Location	-	-	-	-	-
Cyst content	Hydatid sand (*n* = 8) Daughter vesicles (*n* = 29)	1 (1.0) 3 (3.0)	0.50 0.69	-	-

Bold values indicate data from the present study.

### 3.2 Analytical results: predictive factors

Statistical analysis was conducted to identify predictors of specific postoperative complications, with the final results of the regression model presented in Table 5.

**Table 5:** Factors independently associated with specific postoperative complications following radical and conservative surgical treatment of cystic echinococcosis (CE).

Associated factors	*P*-value	OR	95% CI
Intraoperative complications	<0.001	3.51	2.00–10.62
CT-detected complications	0.03	2.59	2.09–20.59

#### 3.2.1 Univariate analysis

Univariate analysis revealed a significant association between preoperative imaging findings and the occurrence of postoperative complications:

- Ultrasound findings: *p* = 0.033.
- CT scan findings: *p* = 0.011.

#### 3.2.2 Multivariate analysis

As shown in Table 5, binary logistic regression identified two independent risk factors significantly associated with an increased risk of specific postoperative complications:

- Preoperative CT scan suspicion of complications: *p* = 0.03.
- Presence of intraoperative complications: *p* < 0.01.

## 4 Discussion

Surgery for hepatic hydatid cysts (HHC) is associated with significant morbidity. In our cohort, univariate analysis identified suspected ultrasound and CT-detected complications as predictive factors for specific postoperative complications. Multivariate analysis confirmed that intraoperative and CT-detected complications were independent predictors of postoperative morbidity. While our study provides valuable insights from a large single-center cohort of 107 patients, its retrospective nature remains a primary limitation.

Biliary fistula is recognized as the most frequent complication of HHC (5). The pathophysiology is multifactorial: as cysts grow—at a rate estimated between 1 and 30 mm per year—intracystic pressure rises, leading to compression, necrosis of the adjacent biliary duct walls, and subsequent fistula formation (2, 6). Conversely, another theory suggests that the entrapment of bile ducts within the cyst wall, combined with high pressure, causes ductal shrinkage followed by cyst wall rupture (6). In the literature, the incidence of biliary fistula ranges from 12 to 37% (Table 6), while large fistulas vary between 4.7% (5) and 25.6% (7). Cyst infection, the second most common complication, ranges from 4.6% (8) to 26.8% (9); our study observed an 11.2% rate.

**Table 6:** Frequency of biliary fistulas across different series.

Author	Year	Total number of patients	Patients with biliary fistulas *n* (%)
Kayaalp (18)	2003	121	45 (37.1)
Hamamci (19)	2005	324	39 (12.0)
Erzurumlu (9)	2005	70	8 (12.0)
Zeybek (20)	2013	282	46 (16.3)
El Malki (14)	2008	649	159 (24.4)
Saylam (20)	2013	135	33 (24.4)
Hailong Lv (21)	2015	153	52 (34.0)
Öztorun (22)	2021	170	8 (4.7)
El Nakeeb (23)	2017	123	21 (17.0)
Our Study	**2025**	**107**	**8 (7.5)**

Bold values indicate data from the present study.

Regarding surgical management, conservative treatment (CT) was utilized in 89.7% of our patients. Although CT is technically less demanding, it is associated with higher morbidity. A meta-analysis by Pang et al. reported a mortality rate of 3.2% and a complication incidence of 41.2% (10). Furthermore, a 2019 meta-analysis (11) of 15 retrospective studies and one randomized trial found no statistically significant difference in mortality between radical and conservative treatments (*p* = 0.91). However, radical treatment (RT) was associated with a lower incidence of postoperative biliary fistula (*p* = 0.00001) and fewer hydatid recurrences (*p* = 0.00001). However, avoiding overly definitive claims regarding superiority is crucial, as the choice of technique depends heavily on individual intraoperative findings. Despite these findings, the recommendation for RT as a standard of care remains supported by low-level scientific evidence.

Predictors of postoperative morbidity in HHC remain poorly established. While several studies (5, 12, 13) suggest that patients over 40 years of age are at higher risk due to comorbidities, our study did not identify age as a significant predictor. Conversely, our findings align with Tunisian research (5) and other studies (12) highlighting that preoperative fever and complications—such as multiple cysts, thick pericysts, and biliary fistulas—significantly increase the risk of postoperative adverse events. Our data confirms a statistically significant association between preoperative CT-detected complex cysts and postoperative morbidity (*p* = 0.03).

While preoperative imaging serves as an essential tool for proactive risk stratification and resource planning, intraoperative complications are inherently realized during the procedure. Consequently, while intraoperative findings are invaluable for immediate intraoperative decision-making and surgical tactical adaptation, they have limited utility for preoperative risk stratification.

From a public health perspective, these findings are crucial. Hydatid disease imposes a substantial burden on healthcare systems, particularly in endemic regions. By identifying high-risk patients through imaging (ultrasound/CT) and intraoperative findings, surgeons can implement proactive management strategies, improve patient triage, and optimize post-surgical surveillance. This targeted approach is essential to reduce the length of hospital stays and the socioeconomic costs associated with complications.

While our study identifies key predictive factors, standardizing the management of complex cysts remains a challenge. We propose that intraoperative ultrasound could be an invaluable tool, particularly in centers with limited expertise in hepatobiliary surgery, to mitigate the risks associated with conservative techniques. Large-scale, randomized trials are warranted to validate these approaches.

Finally, our study is subject to limitations inherent to retrospective research, including potential selection bias and missing data, which may obscure less documented predictive factors. Moreover, the lack of a standardized protocol for selecting between CT and RT may introduce a confounding bias, further emphasizing the urgent need for international guidelines to improve surgical outcomes and reduce the public health impact of this neglected tropical disease.

### 4.1 Strengths, limitations, and recommendations

#### 4.1.1 Strengths

The primary strength of this study lies in its focus on the identification of specific, actionable predictive factors for postoperative morbidity in hepatic hydatid cysts (HHC). By analyzing both preoperative imaging (ultrasound and CT) and intraoperative findings, this study provides a clear risk-stratification model. The inclusion of a cohort of 107 patients in a single-center setting allows for a consistent surgical approach, minimizing inter-operator variability in procedural techniques and postoperative management. Furthermore, our results contribute to filling the gap in the literature regarding the prognostic value of “complex” cyst morphology, providing evidence-based insights that are highly relevant to clinical practice.

#### 4.1.2 Limitations

Despite these findings, several limitations must be acknowledged. Firstly, the retrospective, single-center design is inherently prone to selection bias and potential data loss, which may limit the generalizability of our results. Secondly, the absence of a standardized institutional protocol for selecting between conservative and radical treatments introduces a potential confounding bias, making it difficult to isolate the impact of the surgical technique itself on the incidence of biliary fistulas. Finally, the study was not powered to capture rarer, long-term complications, which are nonetheless significant for patient quality of life.

#### 4.1.3 Recommendations

Based on our findings, we propose the following clinical recommendations to improve surgical outcomes and mitigate the public health burden of HHC:

- Systematic Preoperative Risk Assessment: Surgeons should systematically evaluate preoperative CT imaging for signs of complexity. Identifying “at-risk” cysts should trigger more stringent perioperative monitoring and, potentially, the involvement of a hepatobiliary surgical team.
- Enhanced Perioperative Protocols: Given that intraoperative findings are independent predictors of morbidity, we recommend the mandatory documentation of cyst status and surrounding anatomy during surgery.
- Standardization of Care: Implementing a unified institutional protocol for the selection of surgical techniques (CT vs. RT) is essential to reduce practice variability and improve overall clinical performance.

#### 4.1.4 Perspectives

The management of HHC remains a significant challenge for public health, particularly in endemic regions where the disease impacts both labor productivity and healthcare costs. Future research should prioritize:

- Prospective Randomized Controlled Trials (RCTs): Large-scale, multi-center trials are urgently needed to establish definitive guidelines for the surgical management of complex HHC, moving beyond the current reliance on retrospective evidence.
- Integration of Intraoperative Ultrasound: We recommend further research into the routine use of intraoperative ultrasound to guide surgical decisions, especially in settings with limited specialized hepatobiliary expertise.
- Long-term Socioeconomic Impact Studies: Future studies should measure not only clinical outcomes but also the socioeconomic benefit of reduced postoperative complications, specifically in terms of hospital stay duration and return to work, to inform health policy and resource allocation strategies in endemic areas.

## 5 Conclusion

In conclusion, this study demonstrates that the complexity of hepatic hydatid cysts (HHC), as evidenced by preoperative computed tomography (CT scan) imaging and intraoperative findings, serves as a critical determinant of postoperative morbidity. The integration of these validated predictive markers into clinical protocols represents a robust strategy for optimizing surgical management, enhancing patient stratification, and alleviating the socio-economic and public health burden associated with this condition in endemic regions. Standardizing clinical practice and employing targeted radiological triage are essential steps not only to improve individual surgical outcomes but also to ensure the long-term sustainability of healthcare systems burdened by the persistent impact of cystic echinococcosis.

Beyond clinical risk stratification, how can international public health policies be harmonized to transform these predictive indicators into universal clinical guidelines, while simultaneously ensuring equitable access to specialized hepatobiliary surgical care in regions where this neglected tropical disease continues to strain limited healthcare resources?

## Data Availability

The raw data supporting the conclusions of this article will be made available by the authors, without undue reservation.
